# A novel *RUNX2* splice site mutation in Chinese associated with cleidocranial dysplasia

**DOI:** 10.1016/j.heliyon.2024.e40277

**Published:** 2024-11-08

**Authors:** Jing Wang, Qiuying Li, Hongyu Li, Xiu Liu, Ying Hu, Yuxing Bai, Kai Yang

**Affiliations:** a Department of Orthodontics, School of Stomatology, Beijing Stomatological Hospital, Capital Medical University, No.4 Tiantan Xili, Dong cheng District, Beijing, 100050, China; b Beijing Institute of Dental Research, Beijing Stomatological Hospital, Capital Medical University, Beijing, 100050, China; c Department of Oral Medicine, Beijing Stomatological Hospital, Capital Medical University, Beijing, 100050, China

**Keywords:** Cleidocranial dysplasia, *RUNX2*, Splice-site mutation, DPCR, Chinese population

## Abstract

Pathogenic genes in most patients with cleidocranial dysplasia have been confirmed to be runt-related transcription factor 2 (*RUNX2*), which controls mutations in specific osteoblast transcription factors and affects skull ossification and suture adhesion. This study aimed to explore the role of *RUNX2* mutations. Here, we report a rare case of a splice site mutation in a Chinese population with typical cleidocranial dysplasia symptoms, cranial suture insufficiency, clavicle dysplasia, and dental anomalies. Peripheral blood samples from the proband and her mother were subjected to Sanger sequencing. The expression levels of *RUNX2* before and after mutation were verified using digital PCR (dPCR). The results revealed a classic mutation at the fifth base of the intron 5 initiation splicing sequence (NM001024630.4: C.685+5G > A). The mutation rate in the proband was 53 %, while the mother did not have any mutations. The secondary RNA structure of the *RUNX2* gene in the progenitor was predicted to change, and the structural free energy was low in the wild-type, with the stem folded first and the structure being relatively stable. After the mutation, the free energy increased. This finding enriches the *RUNX2* mutation library of CD-related genes in Chinese individuals.

## 1 Introduction

Cleidocranial dysplasia (CCD), also known as Marie-Sainton syndrome, is a rare congenital genetic disorder with an approximate prevalence of 1:1,000,000 and is characterised by skeletal developmental anomalies, including short stature, fontanel closure insufficiency, clavicular dysplasia, abnormal distal knuckle development, and various dental anomalies [1]. Patients are often treated for dental abnormalities manifesting as retained primary teeth and delayed eruption of permanent or multiple teeth. CCD is an autosomal dominant genetic disorder.

Runt-related transcription factor 2 (*RUNX2*), recognised as a critical determinant in initiating osteoblast lineage commitment, is essential for osteoblast differentiation and osteoprogenitor proliferation [2]. *RUNX2* induces mesenchymal cell proliferation and encourages their transition to osteoblast lineage cells, and more than half of the *RUNX2* gene dosage is required for suturing mesenchymal cells by inducing suture closure. Mutations in the *RUNX2* gene have been implicated in CCD [3]—evidence showing that the main pathogenic gene involved in CCD is *RUNX2*. Deletion, insertion, missense, nonsense, splice-site, and frameshift mutations are the most common types of *RUNX2* mutations reported to cause CCD [4].

The function of *RUNX2* is closely related to the structure of the protein encoded by its mRNA. Different domains of *RUNX2* promote bone reconstruction, including the glutamine/alanine-rich domain (QA), VWRPY region, runt homology domain (RHD), nuclear matrix targeting signal (NMTS), nuclear localisation signal (NLS), proline/serine/threonine rich domain (PST), and repression domain (RD) [5].

Mutations in classical splicing sites can affect transcription and may play an essential role in causing CCD [6]. In the present study, we report a novel classical splicing site mutant of *RUNX2* located at the fifth base of the intron 5 initiator sequence in a patient with a Chinese charge-coupled device (CCD *RUNX2* gene in literature.

## 2 Materials and methods

### 2.1 Patient information

**Fig. 1:**
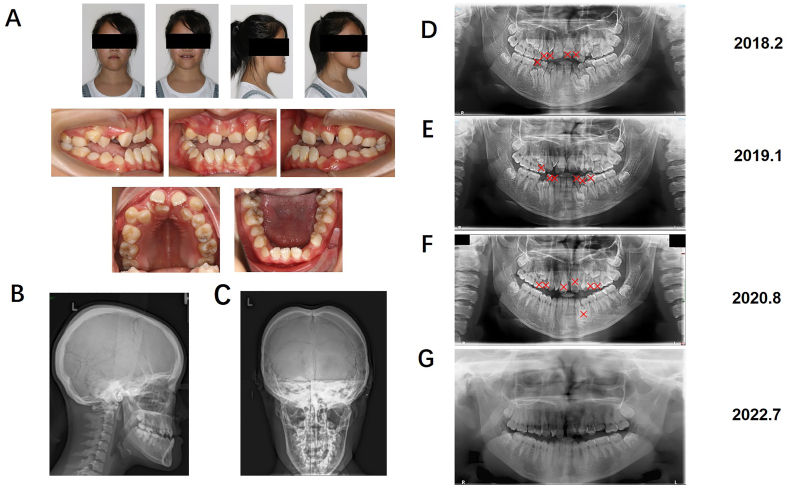
Clinical and radiographic findings of the proband. **A** Frontal bossing in the pretreatment facial photographs and malocclusion in the pretreatment intraoral photographs in 2019.8. **B** Incomplete closure of the cranial suture in the lateral cephalometric radiograph. **C** Skull orthograph showing incomplete closure of the fontanel. Chest radiograph demonstrates a cone-shaped thorax and hypoplasia of clavicles. **(D**–**G)** A panoramic radiograph shows the process of sequential extraction. Each red cross represents a tooth that is scheduled to be removed, including four supernumerary teeth (14 ^P^,11 ^P^,21^P^,34 ^P^) and 14 retained deciduous teeth (52–55, 61–62, 64–65, 73–75, 83–85) (not including the third molars, which had to be followed up on). (For interpretation of the references to colour in this figure legend, the reader is referred to the Web version of this article.)

**Fig. 2:**
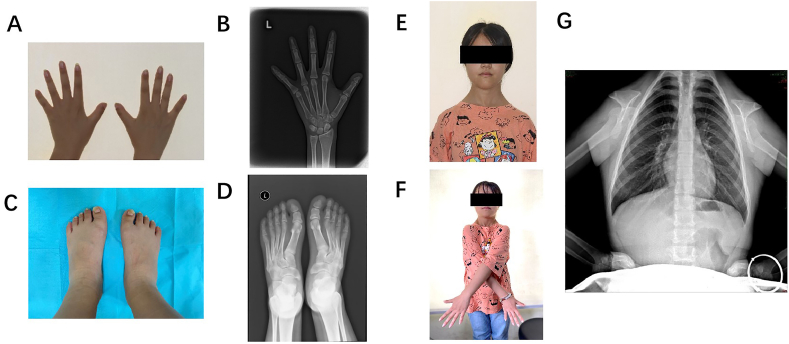
Clinical and radiographic findings of the proband's whole body. Photographing the patient's hand and radiographing the wrist bone (**A** and **B**). Photographs of the patient's feet and foot orthographic radiographs. The distal phalanges of the first toes were shortened, with irregular bony edges (**C** and **D**). Photographs show bilateral deformity and pseudarthrosis of clavicles (**E** and **F**). Plain X-ray of the chest shows bilateral deformity and pseudarthrosis of the clavicles **(G)**.

The proband was an 18-year-old woman admitted to the orthodontic clinic of the Beijing Stomatological Hospital at Capital Medical University with the chief complaints of retention of deciduous teeth and uneven dentition. The patients underwent panoramic radiography or computed tomography (CT) scans for evaluation. The proband was diagnosed with CCD with a typical phenotype, which included a concave face, widened eye distance, mandibular protrusion, typical CCD dental anomalies, such as deciduous tooth retention and redundant teeth (Fig. 1A), short stature, both toes having short joints, narrow shoulders, bilateral deformity, and pseudarthrosis of the clavicles (Fig. 2A, B, C, E, F). Radiographs showed incomplete closure of the fontanel, incomplete closure of the cranial suture, and a protruding mandible (Fig. 1B and C). The distal phalanges of the first toes were shortened with irregular bony edges (Fig. 2D), hypoplastic clavicles (Fig. 2G), and multiple dental anomalies (Fig. 1B). Panoramic radiographs showed that 14 deciduous teeth remained, 17 permanent teeth had not erupted, and there were four superabundant teeth. Based on the development of inherited permanent teeth, crowding of the partial dentition was alleviated by the sequential extraction of the remaining primary and superfluous teeth. In February 2018, the right upper deciduous canine, right upper deciduous incisor, left upper deciduous middle incisor, left upper deciduous incisor, and right lower deciduous second premolar were extracted. In January 2019, the right upper first deciduous molar, left lower deciduous canine, left lower deciduous first and second deciduous molars, and right lower first and second deciduous molars were extracted. Finally, in August 2020, the upper right second deciduous molar and upper left first and second deciduous molars were removed. Four teeth were extracted: one on the palatal side of the first premolar on the upper right side, one on the palatine side of the bilateral maxillary central incisors, and one on the palatine side of the first premolar on the left mandible (Fig. 1D–G). No other signs of intellectual or physical disability were noted. The mother of the proband did not have any characteristics of CCD.

Laboratory tests revealed that although the levels of serum calcium, magnesium, phosphorus, bone alkaline phosphatase, parathyroid hormone, calcitonin, and osteocalcin were within their respective normal ranges, 25-hydroxyvitamin-D3 (25-OH-D3) levels were below normal. Simultaneously, elevated UA levels were observed (Table 1).

**Table 1:** Laboratory results.

Parameter	Results	Unit	Reference range
25-OH-D3 (mg/L)	11.9 (−)	ng/mL	20–60
PTH	23.56	pg/mL	11–62
Calcitonin	3.3	pg/mL	0–50
Osteocalcin	32.2	ng/mL	10–38
Bone AP	77	U/L	43–130
Calcium	2.44	mmol/L	2.1–2.8
Phosphorus	1.28	mmol/L	0.93–1.61
Mg	0.78	mmol/L	0.75–1.02
UA	426.2 (+)	μmol/L	178–416

AP = alkaline phosphatase.

UA = uric acid.

PTH = parathyroid hormone.

Mg = magnesium.

### 2.2 Mutation analysis with Sanger sequencing and identification of variants

Genomic DNA (*g*DNA) was extracted from peripheral blood samples of the patient with CCD and her mother using the Blood Genomic DNA Midi Kit (1–5 mL) (Cwbio, Beijing, China) following the manufacturer's instructions.

Primers for the *RUNX2* gene were designed using Primer3 software (version 4.0.0 (Premier, Canada) (http://bioinfo.utee/primer3/). All exons and exonic–intronic boundary regions of the *RUNX2* gene from the patient and her mother were analysed using polymerase chain reaction amplification and direct Sanger sequencing (Applied Biosystems, Thermo Fisher Scientific, Waltham, MA, USA). Sequence data were analysed using Sequencher software program (version 5.0; Gene Codes Co., Ann Arbor, MI, USA) and sequencing analysis software (version 5.2; ABI, Ann Arbor, MI, USA).

The sequencing results were searched for and compared with information from the Exome Variant Server database. Mutations have not yet been reported in Chinese population databases.

### 2.3 Digital PCR analysis (dPCR)

To validate the mutation's effect, we performed dPCR assays to determine the actual DNA level of RUNX2.

DNA was extracted from the patient's and her mother's peripheral venous blood using the Blood Genomic DNA Midi Kit (1–5 mL) (Cwbio, Beijing, China) according to the manufacturer's instructions. The DNA concentration was determined using a Thermo Scientific NanoDrop 2000/2000c spectrophotometre.

The *RUNX2* primers for dPCR were as follows: forward, 5′TCCCCAAGTAGCTACCTATCACA 3'; reverse, 5′ CCTCATAGGGTCTCTGGAAACTC 3'. The *RUNX2* probes for dPCR were as follows: WT, GTTTCACCTTGACCATAACCGTCTTCACAAATCCTCCCCAAGTAGCTACCTATCACAGAGCAATTAAAGTTACAGTAGATGGACCTCGGGAACCCAGAAGTAAGTACTCCCCTTTTTATTGAAGAAAGTAATAGAGTTTCCAGAGACCCTATGAGGAATTTATTCCAAATGAGTTAGTGT; and mutation, AGTTTCACCTTGACCATAACCGTCTTCACAAATCCTCCCCAAGTAGCTACCTATCACAGAGCAATTAAAGTTACAGTAGATGGACCTCGGGAACCCAGAAGTAAATACTCCCCTTTTTATTGAAGAAAGTAATAGAGTTTCCAGAGACCCTATGAGGAATTTATTCCAAATGAGTTAG. dPCR was performed in a reaction volume of 20 μL containing 10 μL of ddPCR Supermix for Probes (No dUTP), 1 μL of DNA, 3.6 μL of each primer (10 μM), and 1 μL of each probe (10 μM). The reaction was incubated at 95 °C for 10 min, followed by 40 cycles of denaturation at 94 °C for 30 s, annealing at 60 °C for 60 s, and extension at 98 °C for 10 min.

All quantitative data were analysed using a two-tailed Student's t-test using SPSS (version 22.0; IBM Corp., Armonk, NY, USA). Quantitative data were analysed using a two-tailed Student's t-test using SPSS (version 22.0; IBM Corp., Armonk, NY, USA). Each assay was performed in triplicates. Statistical significance was set at P < 0.05.

### 2.4 Predictions of the RNA secondary structure

The mutant *RUNX2* RNA secondary structure was analysed using the RNAup Web Server software. Known intron 5 and exon 5 base sequences of the *RUNX2* FASTA sequences were used as templates for *RUNX2*.

### 2.5 Retrospective study

We have summarised the reported splice site mutations in the *RUNX2* gene literature. All related CCD references were searched for splice site mutations in patients with CCD. The following keywords were used to search PubMed's associated references (published from 1991 to the present): ‘cleidocranial dysplasia’’, splice site mutation,’ and *RUNX2*’. A total of 50 articles that matched the search criteria were retrieved from PubMed; however, only references describing the splice site mutation of *RUNX2* were included in our analysis.

## 3 Results

### 3.1 Sanger Sequencing to determine mutation

**Fig. 3:**
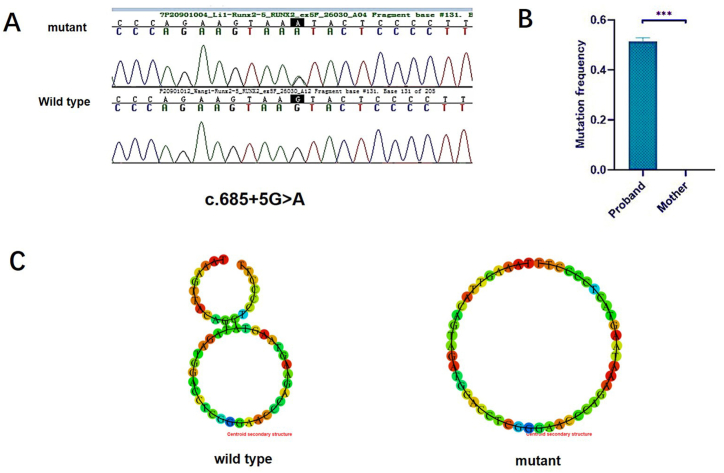
*RUNX2* mutation validation. **A** Sanger Sequencing shows that a splice site mutation in intron 5 of *RUNX2* (NM_001024630.4: c.685+5G > A) was detected in the proband but not in the phenotypically normal mother. **B** The mutation frequency of the *RUNX2* gene in the proband was 53 %, while that in her mother was 0 %. **C** The secondary structure of RNA was predicted using the RNAup Web Server software. The wild type was a more stable stem-loop structure, where the stem with the lowest structural free energy folds first. In the mutation, the structure was a single chain. ∗∗∗ indicates *p* < 0.001 in B.

After filtering the fundamental bioinformatic analysis of the raw exon sequence data for *RUNX2* genes obtained from the proband and her mother and screening in the eRAM (Encyclopedia of Rare Diseases in Asia and the Middle East) database, we identified a previously unreported splice site mutation in the *RUNX2* gene among the Chinese population. More specifically, this mutation manifested as a classical heterozygous change (c.685+5G > A) in intron 5 of *RUNX2* (NM_001024630.4), which was present in the proband but absent in the phenotypically normal mother. Further investigation showed that the mutation site was not present in the patient's father (sequencing results showed that the father's *RUNX2* gene sequence was GGAACCCAGAAGTAAGTACTCCCCTTTTTATTG, with no mutation), indicating that the patient was likely to be a sporadic case (Fig. 3A).

Our findings were prompted by a literature review revealing that patients with CCD often harbour mutations in *RUNX2* gene. Therefore, we designed primers to target all exons of these two genes and performed Sanger sequencing of the corresponding *RUNX2* complementary DNAs (cDNAs) from the proband and her mother. This targeted sequencing effort confirmed the presence of exome sequencing variants initially detected on the Exome Variant Server, with particular emphasis on a novel splice site mutation located in intron 5 of *RUNX2* (NM_001024630.4: c.685+5G > A), exclusive to the proband and hitherto undocumented in the Chinese population within the available literature.

### 3.2 RUNX2 expression analysis

To further investigate the effects of this mutation, dPCR was performed to determine the expression levels of *RUNX2*. Digital PCR results indicated that *RUNX2* levels were significantly downregulated in the proband compared to normal controls. Specifically, the proband exhibited a 53 % decrease in wild-type *RUNX2* levels, resulting in CCD; meanwhile, her mother had hardly altered levels compared to the wild-type control. In other words, the mutation frequency of the *RUNX2* gene in the proband was 53 %, a heterozygous mutation, whereas that in the mother was 0 % (Fig. 3B).

### 3.3 Bioinformatics analysis

We used the RNAup Web Server software program to predict the effect of splice-site mutations on the secondary structure of RNA. The RNA secondary structures of the wild-type and mutant *RUNX2* were markedly different. The gradual accumulation in stems determines the secondary structure of RNA. The stem with the lowest structural free energy folds first, and the free energy value of the wild-type is −5.4. After mutation, the secondary structure is a little less stable and exhibits a slightly reduced free energy value of −5.1 (Fig. 3C).

## 4 Discussion

Approximately 60%–70 % of reported CCD patients with a clinical diagnosis harbour mutations in *RUNX2* [7,8]. *RUNX2* mutations include nonsense, splicing, insertion, microdeletion, missense, microduplication, and frameshift mutations. To date, more than 200 *RUNX2* mutations have been identified, including approximately 5.14 % splicing mutations [9,10].

A classic splicing site mutation is that during the splicing process, the base sequence of the region connecting introns and exons is exon + GT + intron + AG, and mutations at specific sites of GT or AG lead to splicing abnormalities [11]. The patient reported in this paper harboured a mutation at site G at the fifth base of the initial splicing sequence of intron 5 to A, which is a typical splice-site mutation.

After mutation, introns cannot be cut during the subsequent splicing process, eventually leading to abnormalities in the coding sequence (CDS) region of the gene [12], thus affecting the transcription process, which may produce a truncated, non-functional RUNX2 protein, or face degradation due to premature termination codon, therefore significantly reducing the output of the functional protein. In addition, these mutations can affect adjacent regulatory elements such as promoters and enhancers, alter transcription factor binding, impede RNA polymerase II recruitment and transcriptional initiation [13], and ultimately lead to reduced protein synthesis. In addition, their atypical structures can activate cellular RNA quality control mechanisms, further compromising mRNA stability. We also collected *RUNX2* splice site mutations from other reported CCD cases summarised in Table 2.

**Table 2:** Reports of *RUNX2* splice site mutation associated with CCD in the medical literature.

Number	Nucleotide change	Methods	Location	Structural domain	Short stature	Brachycephaly	Delayed closure of fontanels	Frontal bossing	Supernumerary teeth	Clavicular hypoplasia	Vertebral alteration (scoliosis)	References
						
1	c.860-2A > G	Sanger sequencing	Intron 6	PST	+	+	+	+	–	+	–	[23]
						
2	delAAGT	Sanger sequencing	IVS4 + 4	Runt	+	+	+	+	–	+	severe	[24]
3	c.581–9 T > G 8-bp ins	Q-PCR	Terminal exon 5	RHD	+	–	–	–	+	+	–	[25]
4	c.423 + 2delT	Exome or genome	Exon 3	Q/A	+	–	–	+	+	+	–	[26]
		Sequencing										
5	IVS2+1G > C	DNA sequencing	Intron 2	Runt	+	+	+	+	+		+	[27]
						
6	IVS1+1G > A	Q-PCR	Exon 1–Intron junction	Q/A, Runt	+	+	+	+	+	+	–	[28]
7	IVS2 + 2 T > A	Q-PCR	Exon 2–Intron junction splice site	Runt	+	+	+	–	+	+	–	[29]
8	IVS6-1 G > C	Q-PCR	Exon 7	Runt	–	+	–	–	+	+	–	[30]
9	IVS3 + 3delAAGT	RT-PCR	Exon 3–Intron junction	Runt	+	+	+	–	+	+	–	[31]
10	c.580 + 1G > A	RT-PCR	Intron 2	Runt	+	+	–	–	+	+	–	[32]
11	c.860 - 2A > G	Sanger sequencing	Intron 5–Exon 6 splice site boundary	Runt and PST	+	–	+	–	+	+	+	[33]
12	IVS3 + 3delAAGT	Exome or genome sequencing	Intron 3	Entire areas	–	–	+	+	+	+	–	[34]

CCD, cleidocranial dysplasia; PST, proline/serine/threonine-rich region; Runt, Runt domain; RHD, Runt homologous domain; Q/A, glutamine–alanine repeat domain; Q-PCR, quantitative polymerase chain reaction; RT-PCR, real-time polymerase chain reaction.

*RUNX2* mutations do not necessarily lead to the clinical symptoms of CCD; therefore, it is essential to calculate the mutation rate of *RUNX2*. A study by Tomimatsu et al. verified that the mRNA level of *RUNX2* in patients decreased by 75.5 %, and significant CCD symptoms occurred [12]. Kim et al. reported that *RUNX2* mRNA levels were reduced by 34.3 % in the proband's sister, who did not have CCD symptoms [13]. In a different report, the mother of one CCD patient did not exhibit any CCD phenotypes but carried approximately 21.8 % of a *RUNX2* gene mutation in exons 1–4 [14]. In our study, the patient had a mutation rate of the *RUNX2* gene of 53 % and showed typical clinical manifestations of CCD. In addition to *RUNX2* expression levels, the type and location of mutations may lead to phenotypic changes, including missense, splice site, nonsense, insertion/deletion, repetition, and frameshift mutations. Mutation sites include QA, VWRPY region, RHD, NMTS, NLS, PST, and RD.

Some biomarkers have essential guiding significance in the diagnosis of various diseases. Serum 25-OH-D3 levels are closely associated with bone mineral content. Decreased 25-OH-D3 levels can lead to osteomalacia, rickets, secondary osteoporotic fractures, and other consequences [15]. In this study, the 25-OH-D3 level in the patient was 11.9 ng/mL, which is significantly lower than the normal range, and this abnormality may be one of the indicators used to evaluate CCD. Animal studies have shown that a high concentration of uric acid in the blood can inhibit 1-α-hydroxylase, which may reduce the level of 25-OH-D3 and ultimately lead to reduced activation of vitamin D [16]. Therefore, uric acid may affect bone formation by inhibiting vitamin D activation, thereby affecting bone health. In our study, the patient's uric acid level was 426.2 μmol/L, being higher than normal, which may be one of the factors affecting bone health in CCD. However, some studies have shown no significant relationship between decreased bone mass and serum 25-OH-D3 level (p > 0.05). Therefore, further exploration of the relationship between serum 25-OH-D3 levels and the CCD phenotype is needed.

In future studies, we will collect data on 25-OH-D3 and uric acid levels in CCD patients at the childhood, adolescence, and adulthood stages of disease development to observe the dynamic relationship between them and disease progression. By analysing how these biomarkers change early in the disease process, we hope to identify patterns that indicate increased disease severity. This is significant for implementing early intervention measures, optimising treatment plans, and improving the patients' quality of life. We conducted longitudinal studies to track the changes in these biomarkers in the same group of patients over time to explore their potential as prognostic indicators.

In this study, we predicted the secondary structure prediction analysis was performed for the first time. In Berkay's study, a mutation at this site in patients with CCD in Turkey was reported, indicating that the genetic background differences of different races did not affect the pathogenesis of CCD. Specific analysis of clinical phenotypes showed that, compared to CCD patients in Turkey, the finger bones of Chinese patients showed a shortened phenotype with irregular bone margins, indicating that even if the mutation was the same, different modifying genes might cause different pathogeneses, further producing phenotypic differences [17]. This is a direction that we need to explore in future studies.

The biological functions of RNA are primarily determined by their secondary structures. The planar structure formed by different single-stranded regions, stem-loop structures, and double-stranded regions forms the secondary structure of RNA through self-folding [18]. The secondary structure of the RNA is the result of stem accumulation. The stem is a collection of two opposing, complementary base pairs [19]. Individual complementary base pairs are generally considered unstable, and complementary base pairs tend to appear in strings. When the free energy is low, and the distance between the pairs of bases is small, the stem is more likely to fold first. The patient had a *RUNX2* free energy of −5.1 after the mutation. In the wild type, *RUNX2* has a complementary base structure in the cluster, lower free energy of −5.4, the stem folds first, and the secondary structure is more stable.

The secondary structure of RNA can change the stability and degradation of RNA, may cause some codons not to be correctly recognised, and may interfere with the folding process of proteins by affecting early ribosome density and translation extension, thereby affecting its binding to the ribosome. Therefore, changes in RNA secondary structure dynamics can alter the function of protein interactions by affecting the total amount, structure, and stability of proteins [[18], [19], [20]]. Changes in the secondary structure of RNA can affect the clinical manifestations of CCD by interfering with osteogenic differentiation, cell cycle regulation, apoptosis, other cell signalling pathways, and epigenetic regulation [5].

To verify the accuracy of Sanger sequencing results, we used absolute quantitative dPCR, also known as single-molecule PCR. In contrast to traditional techniques, dPCR generally requires the dilution of the sample to the single-molecule level in the amplification stage and even distribution to tens of thousands of reaction units [21]. After amplification, the fluorescence signal of each reaction unit was collected, and the original sample content was obtained by direct counting. The advantage of the quantitative dPCR method is that it does not depend on the cyclic threshold of the amplification curve and does not require the use of steward genes and standard curves. It demonstrated commendable accuracy and reliability, making an absolute quantitative evaluation possible [22].

In our study, there was only one such case. In future studies, we will conduct cell and animal experiments to overcome the limitations of our single case by designing a mutant and performing functional analysis at the cellular and molecular levels to verify the relationship between the disease and the mutation site. We plan to expand the sample size and number of families to supplement the results and conclusions of this study.

In a future study on CCD, we will use CRISPR-Cas9 gene editing technology to explore the possibility of correcting CCD gene mutations. In addition, it is critical to identify and validate novel therapeutic targets for CCD, including bone metabolic modulators, signalling pathway inhibitors or promoters, and microenvironmental factors that influence osteogenesis and cartilage formation [22]. Given the genetic heterogeneity of CCD, it is essential to develop personalised precision medicine. This includes building patient databases and biobanks to support the development of big data analytics and machine learning algorithms for predicting disease progression, treatment response, and potential complications [23,24]. In addition, attention should be paid to the impact of CCD on patients' self-esteem, social skills, and mental health to improve their quality of life.

## 5 Conclusion

This paper reports a case of cleidocranial dysplasia in China. The pathogenic site is a classic mutation at the fifth base of the intron 5 initiation splicing sequence in the *RUNX2* gene (NM001024630.4: C.685+5G > A). This mutation led to changes in *RUNX2* gene expression levels in this patient, further affecting bone development and function. In addition, by comparing the changes in the RNA secondary structure before and after mutation, we speculated that mutations may affect protein function by altering mRNA stability and translation efficiency.

Overall, this study enriches our understanding of CCD pathogenesis and provides new ideas for a more precise treatment of the disease.

## CRediT authorship contribution statement

**Jing Wang:** Writing – review & editing, Writing – original draft, Funding acquisition, Data curation, Conceptualization. **Qiuying Li:** Writing – original draft, Investigation. **Hongyu Li:** Writing – original draft, Supervision, Methodology. **Xiu Liu:** Writing – original draft, Visualization, Data curation. **Ying Hu:** Writing – original draft, Validation. **Yuxing Bai:** Writing – original draft, Project administration. **Kai Yang:** Writing – review & editing, Validation, Project administration, Funding acquisition, Conceptualization.

## Consent to participate

Informed consent was obtained from all individual participants included in the study.

## Consent to publish

Informed consent was obtained from all the participants.

## Ethical statement

This study was approved by the Institutional Review Board of Beijing Stomatological Hospital, Capital Medical University (approval no. CMUSH-IRB-KJ-PJ-2023-07). Written informed consent was obtained from each participant to publish any potentially identifiable images or data included in this article.

## Data availability statement

The data have been deposited at [Mendeley data] with the accession number [kwwjjxa@163.com].

## Funding

We appreciate the project being supported by the Beijing Stomatological Hospital, 10.13039/501100002799Capital Medical University Young Scientist Program, No. YSP202110 (JW (Jing Wang)), 10.13039/100016126Beijing Hospital Authority Ascent Plan, No. DFL20191501 (KY), Beijing Natural Science Foundation [Grant Number 7222077] (KY), and the 10.13039/501100001809National Natural Science Foundation of China (Grant No.81771103) (KY). We are grateful to the proband and his family for participating in this study.

## Declaration of competing interest

The authors declare that they have no known competing financial interests or personal relationships that could have appeared to influence the work reported in this paper.
